# Developmental dynamics of ovine lung in health and cystic fibrosis at single-cell resolution

**DOI:** 10.1007/s10142-026-01981-2

**Published:** 2026-07-31

**Authors:** Svyatoslav Tkachenko, Shih-Hsing Leir, Arnaud J. Van Wettere, Iuri Viotti Perisse, Yasmin Mustafa, Cheyenne M. Marriott, Tayler Patrick, Ying Liu, Kenneth L. White, Irina A. Polejaeva, Ann Harris

**Affiliations:** 1 Department of Genetics and Genome Sciences, Case Western Reserve University, Cleveland, OH USA; 2 Department of Veterinary Clinical and Life Sciences, Utah State University, Logan, UT USA; 3 Department of Animal, Dairy and Veterinary Sciences, Utah State University, Logan, UT USA

**Keywords:** Lung development, ScRNA-seq, Cystic fibrosis (CF), *CFTR*, Sheep

## Abstract

The sheep lung has long been used to model aspects of the human lung, both its normal development and pathological changes that occur in ovine models of lung disease. The similarities between the two species provide an opportunity to investigate phases of lung development that are not accessible for investigation in humans, for example later in gestation. Here we use single cell (sc) RNA-seq to investigate the developmental dynamics of the sheep proximal and distal lung from mid gestation (80 days) through late gestation (120 days) and term (147 days). We identify changes in cell identity and abundance between the time points that illustrate many key features of mesenchymal, endothelial, and epithelial differentiation. Lastly we compare the single cell profiles through development of wildtype (WT) sheep with those of animals of the same breed engineered to have a loss-of-function mutation in the cystic fibrosis transmembrane conductance regulator (*CFTR*) gene. Mutations in *CFTR* cause cystic fibrosis (CF) and the *CFTR*^*−/−*^ sheep exhibit many features of CF in humans. The results identify cellular signatures in the *CFTR*^*−/−*^ lung before birth that may facilitate the understanding of clinical features of CF in early postnatal life.

**Supplementary Information:**

The online version contains supplementary material available at 10.1007/s10142-026-01981-2.

## Introduction

Many aspects of human fetal life are not accessible for study, hence much information is based on the investigation of other mammals. Over many decades, the sheep has been used as a comparative model for human fetal ontogeny, in part due to the similarities in placental maturation, metabolic function and nutrient transport (Barry and Anthony 2008). Also, human and sheep exhibit similar lung development, size and physiology (Scheerlinck et al. 2008; Meeusen et al. 2009), numbers of offspring and a longer gestation (~ 147 days in sheep, 280 days in human). In part due to these similarities sheep are a preferred large animal model for research into human lung diseases with prenatal or postnatal origins. We developed sheep models of cystic fibrosis (CF) (Fan et al. 2018; Viotti Perisse et al. 2021; Van Wettere et al. 2022) to investigate early developmental aspects of this common inherited disorder, which impairs multiple organs including those within the respiratory and digestive systems.

More recently, we used these models to examine the developmental time course of the lung, pancreas, liver and gallbladder in WT and CF sheep (Kerschner et al. 2023; Leir et al. 2024, 2025; Meckler et al. 2025). Applying genome-wide technologies (bulk RNA-seq and single cell (sc) RNA-seq) at specific time points through sheep gestation we observed differences between the transcriptomes of CF and WT animals that reflected pathological changes in the CF organs. In the CF lung, a transient inflammatory signature between 100 and 120 days (Kerschner et al. 2023) preceded a cellular imbalance at term. Ciliated cells were overabundant in the CF sheep in both proximal and distal lung at term, while secretory epithelial cells and basal cells were underrepresented in proximal lung as were T cells and monocytes in the distal lung (Leir et al. 2025).

Our previous analysis of the scRNA-seq data from the lung developmental time course focused primarily on the disease-associated features of the CF fetuses. Here, we have used these data instead to learn about the developmental dynamics of different cell lineages in the WT and CF proximal and distal lung. These analyses are particularly informative for later gestational time points, which are not available for human fetal development. Recent comprehensive scRNA-seq analysis of human fetal lung tissues until 22 weeks have provided extensive datasets for comparative genomics, and provided remarkable precision on cell identities, locations in the developing lung and developmental trajectories (He et al. 2022; Sountoulidis et al. 2023; Cao et al. 2023). Similarly, multiple datasets incorporating single cell transcriptomics with spatial and temporal information in the adult human lung, in both health and disease, continue to refine our understanding of lung biology (Montoro et al. 2018; Plasschaert et al. 2018; Deprez et al. 2020; Travaglini et al. 2020; Sikkema et al. 2023; Carraro et al. 2021; Firsova et al. 2025; Basil et al. 2022; Vieira Braga et al. 2019). However, there remains a temporal knowledge gap between the human fetal lung at 22 weeks and the post-natal lung datasets.

Human lung development is divided into five overlapping developmental stages defined largely on anatomical transitions and measured in post-conception weeks (pcw) (Burri 1984; Nikolic et al. 2018). During the embryonic stage (4–7 pcw), the bilateral lung buds arise from the foregut endoderm and their rapid branching between 4 and 5 weeks creates the lobular structure of the lung. Branching morphogenesis continues through the pseudoglandular stage (5–17 pcw) during which cartilaginous airways develop. Further epithelial branching and differentiation coincides with blood vessel development. The canalicular phase (16–26 pcw) is marked by continued airway growth, additional epithelial branching and generation of the future alveolar regions. Small blood vessels become closely associated with the distal airspaces, where alveolar epithelial differentiation commences. The small airways terminate in thin-walled saccules at the start of the saccular stage (24–38 pcw), during which the respiratory airspaces undergo major expansion and interstitial tissues diminish. Concurrently, the capillary network envelops the saccules within which alveolar epithelial differentiation continues and is marked by the start of surfactant production. Lastly, during the alveolar stage (36 pcw to 3 years), saccules become divided into alveoli as septa develop from the saccular walls, enabling the large increase in surface area that is required for gas exchange. At the same time, capillary differentiation establishes the network required to support these surfaces. This time course illustrates the substantial cellular and functional differentiation that occurs after the pseudoglandular stage and is critical for normal lung function.

Currently available human fetal lung single cell data include tissues from 4 to 8 pcw (Cao et al. 2023), 5 to 14 weeks (Sountoulidis et al. 2023), 5 to 22 weeks (He et al. 2022) and 11.5 to 21 weeks (Miller et al. 2020). Hence, the focus is on the embryonic and pseudoglandular stages of human lung development, but much less is known about the cellularity and developmental trajectories through the canalicular, saccular and prenatal alveolar stages.

The sheep lung has a similar, though not identical, developmental time course to the human lung. Based upon the different durations of gestation, we previously correlated the two species (Kerschner et al. 2023) as a foundation for the choice of time intervals in a transcriptomic analysis of sheep lung development. In bulk RNA-seq analysis we chose 50-, 65-, 80-, 100-, 120- and 147 (term) days of sheep gestation for tissue collection, thus spanning pseudoglandular, canalicular, saccular, and alveolar stages of development. For scRNA-seq analysis the time points were fewer at 80-, 120- and 147 days (late pseudoglandular to alveolar) (Leir et al. 2025). Here we use simple comparative protocols to examine the alterations in cell type abundance between 80 and 120 days, and between 120 days and term in sheep proximal and distal lung. These results provide real time analysis to complement previous pseudotime predictions across a poorly studied window of lung development.

## Methods

The materials and methods for this study were as described in our previous work (Van Wettere et al. 2022; Kerschner et al. 2023; Leir et al. 2024, 2025), but are briefly reiterated here.

### Sex as a biological variable

Our study examined male and female animals where possible since all cloned animals used here are male (Leir et al. 2025). Results are combined and sex was not considered as a biological variable.

### Animals

American Romney breed of domestic sheep (*Ovis aries*) was used in this study. All animal studies were approved and monitored by the Institutional Animal Care and Use Committee (IACUC) at Utah State University (IACUC protocol # 10089) and conformed to the National Institute of Health guidelines. WT sheep were bred according to standard protocols and are summarized in Table 1. *CFTR*^*−/−*^ sheep pregnancies were generated by somatic cell nuclear transfer (SCNT) or by natural breeding as shown in Table 2.

**Table 1:** Wild type samples for scRNA-seq analysis

Age	Distal/Proximal	scRNA-seq ID/(cell #)	Animal #	Genotype	Sex
80d	Proximal	AXH035 (3163)	SF1501-1	WT	M
80d	Proximal	AXH063 (411)	SFWT194-1	*CFTR*^+/-^	M
80d	Proximal	AXH064 (2169)	SFWT194-2	WT	M
80d	Proximal	AXH065 (1782)	SFWT194-3	*CFTR*^+/-^	M
120d	Proximal	AXH043 (3575)	SF1301-1	WT	M
120d	Proximal	AXH044 (4104)	SF1301-2	WT	F
120d	Proximal	AXH071 (11,031)	SFWT1601-2	WT	M
120d	Proximal	AXH072 (9810)	SFWT1601-3	WT	M
term 72 h	Proximal	AXH021 (2690)	WT2004	WT	M
term 6 h	Proximal	AXH038 (2761)	SF173-1	WT	M
term 14 h	Proximal	AXH047 (6710)	2298	WT	F
term 5.5 h	Proximal	AXH138 (4770)	SFWT2281	WT	F
term 62 h	Proximal	AXH141 (4706)	SFWT1615	WT	M
80d	Distal	AXH034 (3532)	SF1501-1	WT	M
80d	Distal	AXH060 (2461)	SFWT194-1	*CFTR*^+/-^	M
80d	Distal	AXH061 (1984)	SFWT194-2	WT	M
80d	Distal	AXH062 (590)	SFWT194-3	*CFTR*^+/-^	M
120d	Distal	AXH041 (3209)	SF1301-1	WT	M
120d	Distal	AXH042 (3052)	SF1301-2	WT	F
120d	Distal	AXH069 (8983)	SFWT1601-2	WT	M
120d	Distal	AXH070 (7754)	SFWT1601-3	WT	M
term 72 h	Distal	AXH022 (2273)	WT2004	WT	M
term 6 h	Distal	AXH037 (2900)	SF173-1	WT	M
term 14 h	Distal	AXH046 (10,012)	2298	WT	F
term ~ 16 h	Distal	AXH089 (11,932)	SFWT2011-1	WT	M
term 5.5 h	Distal	AXH137 (4172)	SFWT2281	WT	F
term 62 h	Distal	AXH140 (4296)	SFWT1615	WT	M

**Table 2:** *CFTR*^*−/−*^samples for scRNA-seq analysis

Age	Distal/Proximal	scRNA-seq ID/(cell #)	Animal #	Genotype	Sex	Cloned/Natural
80d	Proximal	AXH058 (2941)	SFY1722-1	*CFTR*^*−/−*^	M	cloned
80d	Proximal	AXH119 (3679)	SFP2050-1	*CFTR*^*−/−*^	M	cloned
80d	Proximal	AXH120 (8681)	SFP2050-2	*CFTR*^*−/−*^	M	cloned
120d	Proximal	AXH077 (7172)	SFY1705A^*^	*CFTR*^*−/−*^	M	cloned
120d	Proximal	AXH078 (7553)	SFY1705B^*^	*CFTR*^*−/−*^	M	cloned
120d	Proximal	AXH125 (4458)	SFY1905-1	*CFTR*^*−/−*^	M	cloned
120d	Proximal	AXH126 (3864)	SFY1905-2	*CFTR*^*−/−*^	M	cloned
120d	Proximal	AXH129 (3629)	SFP2077	*CFTR*^*−/−*^	M	cloned
120d	Proximal	AXH134 (4537)	SFCF2502-1	*CFTR*^*−/−*^	F	natural
120d	Proximal	AXH135 (3895)	SFCF2502-2	*CFTR*^*−/−*^	F	natural
term 18 h	Proximal	AXH053 (5978)	CF2504	*CFTR*^*−/−*^	F	natural
term 60 h	Proximal	AXH086 (3051)	CF2103	*CFTR*^*−/−*^	F	natural
term ~ 16 h	Proximal	AXH111 (4087)	SFCF2114	*CFTR*^*−/−*^	F	natural
term 9 h	Proximal	AXH146 (5142)	SFP2058A^*^	*CFTR*^*−/−*^	M	cloned
term 9 h	Proximal	AXH147 (6450)	SFP2058B^*^	*CFTR*^*−/−*^	M	cloned
80d	Distal	AXH057 (2107)	SFY1722-1	*CFTR*^*−/−*^	M	cloned
80d	Distal	AXH117 (3346)	SFP2050-1	*CFTR*^*−/−*^	M	cloned
80d	Distal	AXH118 (3123)	SFP2050-2	*CFTR*^*−/−*^	M	cloned
120d	Distal	AXH075 (10,137)	SFY1705A^*^	*CFTR*^*−/−*^	M	cloned
120d	Distal	AXH076 (9983)	SFY1705B^*^	*CFTR*^*−/−*^	M	cloned
120d	Distal	AXH123 (3232)	SFY1905-1	*CFTR*^*−/−*^	M	cloned
120d	Distal	AXH124 (3516)	SFY1905-2	*CFTR*^*−/−*^	M	cloned
120d	Distal	AXH128 (3860)	SFP2077	*CFTR*^*−/−*^	M	cloned
120d	Distal	AXH132 (5424)	SFCF2502-1	*CFTR*^*−/−*^	F	natural
120d	Distal	AXH133 (5829)	SFCF2502-2	*CFTR*^*−/−*^	F	natural
term 18 h	Distal	AXH050 (12,844)	CF2503	*CFTR*^*−/−*^	M	natural
term 18 h	Distal	AXH051 (9068)	CF2504	*CFTR*^*−/−*^	F	natural
term 60 h	Distal	AXH085 (4643)	CF2103	*CFTR*^*−/−*^	F	natural
term ~ 16 h	Distal	AXH110 (3623)	SFCF2114	*CFTR*^*−/−*^	F	natural
term 9 h	Distal	AXH144 (5288)	SFP2058A^*^	*CFTR*^*−/−*^	M	cloned
term 9 h	Distal	AXH145 (5328)	SFP2058B^*^	*CFTR*^*−/−*^	M	cloned

Key: ^*^denotes 2 separate samples from one animal

### Histopathologic analysis

A necropsy was performed on all fetuses collected, to examine for gross lesions and the findings were documented as described previously (Van Wettere et al. 2022). Lung tissue samples were collected and fixed in 10% neutral buffered formalin for histology.

### Single-cell RNA sequencing (scRNA-seq)

#### Isolation of single cells from tissues

Tissues for scRNA-seq were from WT animals at 80-, 120- and 147 days (term) gestation, cloned *CFTR*^*−/−*^ animals at 80-, 120-days and term, and naturally bred *CFTR*^*−/−*^ lambs at 120-days and term. Proximal and distal lung regions for single cells isolation are shown in Fig. S1 and detailed protocols are in (Leir et al. 2025).

#### Single-cell RNA-sequencing and analysis

Approximately 3000- 5000 cells were used for scRNA-seq using the 10 × Genomics Chromium Single Cell 3’ Reagent Kit v3, or v3.1. The libraries were sequenced on a NovaSeq 6000 machine. Reads were aligned to the Oar_v4.0/oviAri4 (Texel) genome using Cell Ranger 3.1.0. Cells were filtered for quality using cuts for standard metrics: library size, number of detected genes and mitochondrial read percentage. Ribosomal protein genes were also filtered out before the generation of Seurat objects from the QC filtered files. The resulting objects were normalized and batch-corrected using the Seurat R package (Hao et al. 2021), version 5.1.0, followed by clustering and UMAP (Uniform Manifold Approximation and Projection) dimensionality reduction. Seurat was also used to find cluster markers by performing differential gene expression analysis between clusters using the receiver operating characteristic (ROC) method. To compare cell proportions between time point (120 days vs 80 days, or term vs 120 days) in each cluster, a Monte-Carlo/permutation test was performed using the scProportionTest R package (https://github.com/rpolicastro/scProportionTest) (Miller et al. 2021). Separate analyses were performed in proximal and distal lung in WT and *CFTR*^*−/−*^ animals.

#### Detailed parameters of scRNA-seq analysis

*Normalization* of single-cell data was performed utilizing the Seurat NormalizeData function with the default ‘LogNormalize’ method: Feature counts for each cell were divided by the total counts for that cell and multiplied by the empirical ‘scale.factor’, for which the default value of 10,000 was used. This was then natural-log transformed using ‘log1p’.

*Variable features* of the single-cell data were found using Seurat FindVariableFeatures function with the default ‘vst’ selection method: a line was fitted to the relationship of log(variance) and log(mean) using local polynomial regression. Then, the feature values were standardized using the observed mean and expected variance (given by the fitted line). Feature variance was then calculated on the standardized values after clipping to a maximum (a default value was used equal to the square root of the number of cells).

*Features for integrating samples* were found using the Seurat SelectIntegrationFeatures function with the default number of used features (2000): features were ranked by the number of datasets they were deemed to be variable in, breaking ties by the median variable feature rank across datasets. 2000 top scoring features were returned by this ranking.

*Sample integration* was performed using the Seurat IntegrateData function with default parameters, with anchors for integration found by running the Seurat SelectIntegrationFeatures function with default parameters.

*PCA dimensionality reduction* was performed using the Seurat RunPCA function with default parameters using variable features found by the FindVariableFeatures function.

*Clustering*: the dataset was clustered by first running the Seurat FindNeighbors function with the number of dimensions found from the elbow plot, followed by the Seurat FindClusters command that identifies clusters of cells by a shared nearest neighbor modularity optimization based clustering algorithm. A resolution of 0.2 was used unless stated otherwise.

*Uniform Manifold Approximation and Projection (UMAP)* dimensional reduction was performed using the Seurat RunUMAP function with the number of dimensions found from the elbow plot.

*Cluster markers* were found by running the Seurat FindAllMarkers function using the ROC method. This identifies ‘markers’ of gene expression using ROC analysis. For each gene, ROC evaluates—using AUC – a classifier built on that gene alone, to classify between two groups of cells. Genes expressed in minimum of 5% of cells in either of the two compared populations and showing at least a 0.25 log-scale difference between the two groups were recorded. Only positive markers were saved.

A proportions test was used to measure the asymmetry in cell numbers of specific kinds between populations, and was performed using a permutation test as presented in the permutation_test function from the scProportionTest package. The default number of permutations (1000) was used.

## Results

The identification of cell clusters was based on our previous detailed scRNA-seq analysis of marker genes in the sheep fetal lung (Leir et al. 2025). We chose a resolution that enabled full cluster identification rather than one which generated a higher number of clusters.

### Evolution of cell type abundance through gestation in WT proximal lung

#### A. Proximal lung development between 80 and 120 days of gestation coincides with an increased abundance of basal cells, ciliated cells and endothelial cell types

**Fig. 1:**
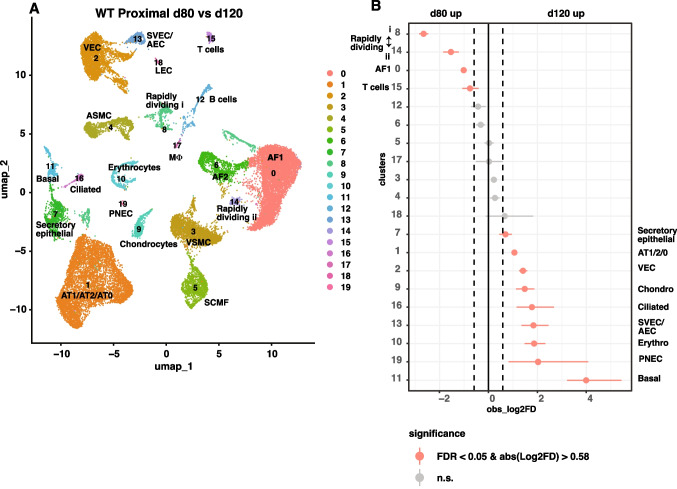
Single cell RNA-seq shows developmental changes in cell abundance between 80 and 120 days in WT sheep proximal lung. **A**. UMAP plot of merged data from 8 donors (4 each at 80 days and 120 days) and identifies 20 clusters by differential gene expression profiles, each named by cell type. Abbreviations: AF1/2, alveolar fibroblasts (1/2); AT1/2/0, alveolar type 1/2/0; VEC, vascular endothelial cells; VSMC, vascular smooth muscle cells; ASMC, airway smooth muscle cells; SCMF, secondary crest myofibroblast; Chondro, chondrocytes; SVEC, systemic venous endothelial cells; AEC arterial endothelial cells; MΦ, macrophages; LEC, lymphatic endothelial cells; PNEC, pulmonary neuroendocrine cells. **B**. Single cell proportions test comparing day 80 to day 120 shows significant overrepresentation of cells in 80 day clusters on the left and in 120 day clusters on the right

Comparing 80 day and 120 day proximal WT lung at a resolution of 0.2, we identified 20 cell clusters based on marker gene profiles (Fig. 1A, Table S1). At 80 days, some of the cell identities, particularly mesenchymal cells were challenging to assign due to the presence of immature cells expressing diverse markers. Considering clusters according to their development origin: 1) Mesoderm-derived: clusters 0, 3, 4, 5, and 6 are all mesenchymal cells; clusters 2, 13 and 18 are endothelial cells; clusters 12, 15 and 17 are immune cells; cluster 9 are chondrocytes; and cluster 10 are erythrocytes. 2) Endoderm-derived: clusters 7, 11 and 16 are epithelial cells; and cluster 19 pulmonary neuroendocrine cells (PNEC). Ectoderm-derived neuronal cells were rare in our analysis. The identity of each cluster based on marker genes defined in our earlier work (Leir et al. 2025) is shown in Fig. 1A. We compared the proportions of 80 day and 120 day cells contributing to each cluster (Fig. 1B), with clusters that were more abundant in 80 day WT proximal lung on the left of the plot (0) and those on the right more abundant at 120 days. Key marker genes for identifying each cluster, referred to by their official gene symbol are shown in Table Si, with full gene names in Table Sii. The few clusters that were significantly more abundant at 80 days included: rapidly dividing cells (cluster 8), identified by many genes involved in the cell cycle/cell division, and others involved in DNA replication, though with no clear markers of differentiated lung cell types; another cluster (14) of cells with some markers of alveolar fibroblasts (AF1 cells) but a predominance of genes involved in cell proliferation, suggesting that these are rapidly dividing AF1-like cells; cluster 0, which have characteristic markers of AF1 cells; lastly cluster 15, immune cells which are likely predominantly T cells. In contrast, more (nine) clusters are significantly more abundant in 120 day WT proximal lung including: basal cells (cluster 11); PNEC (cluster 19, albeit with a large error bar in Fig. 1B); erythroid cells (cluster 10); systemic venous endothelial cells (SVEC) or arterial endothelial cells (AEC), (cluster 13); ciliated cells (cluster 16); chondrocytes (cluster 9); vascular endothelial cells (VEC) (cluster 2); and secretory epithelial cells (cluster 7), which just reaches statistical significance. Of note the abundance of erythroid cells reflects the efficacy of their depletion during the single cell preparation protocol, rather than being a primary feature of the tissue samples. The remaining six clusters including cluster 12 (B cells), cluster 6 (AF2 cells), cluster 5 (secondary crest myofibroblasts (SCMF)), cluster 17 (macrophages), cluster 3 (vascular smooth muscle cells (VSMC)), cluster 4 (airway smooth muscle cells (ASMC)), and cluster 18 (lymphatic endothelial cells (LEC)) were equally abundant at 80 and 120 days.

In summary, the major changes in cell abundance between 80 and 120 days in proximal lung are i) a reduction in populations of poorly differentiated rapidly dividing cells and fibroblasts, and ii) an increase in multiple differentiated epithelial cell types and also in several endothelial cell populations. These observations are consistent with the late pseudoglandular stage of development in the human lung, during which cartilaginous airways develop and ongoing epithelial branching and differentiation coincides with blood vessel amplification.

#### B. Proximal lung development between 120 days of gestation and term coincides with an increased abundance of specific epithelial cells, immune cells and endothelial cell types

**Fig. 2:**
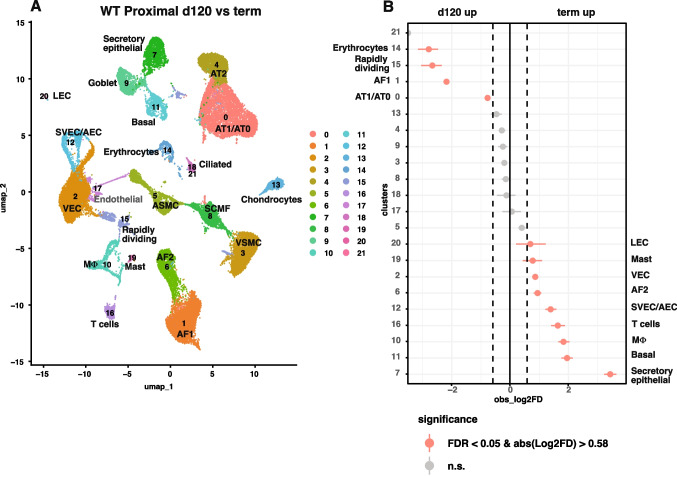
Single cell RNA-seq shows developmental changes in cell abundance between 120 days and term in WT sheep proximal lung. **A**. UMAP plot of merged data from 9 donors (4 at 120 days and 5 at term) and identifies 22 clusters by differential gene expression profiles, each named by cell type. Abbreviations as in Fig. 1 legend. **B**. Single cell proportions test comparing day 120 to term shows significant overrepresentation of cells in 120 day clusters on the left and in term clusters on the right

We next compared 120 day and term proximal WT lung at a resolution of 0.2 and identified 22 cell clusters based on marker gene profiles (Fig. 2A, Table S2). Clusters 1, 3, 5, 6, and 8 are all mesenchymal cells, clusters 2, 12 and 20 are endothelial cells, clusters 10, 16 and 19 are immune cells and cluster 14 are erythrocytes. Endoderm-derived cell clusters include clusters 7, 9, 11, 13 and 18 epithelial cell clusters. The identity of each cluster is again based on marker genes defined in our earlier work (Leir et al. 2025) and is shown in Fig. 2A and Table Si and Sii. The single cell proportions test comparing 120 day and term proximal lung is shown in Fig. 2B, with clusters that were more abundant at 120 days on the left of the plot (0) and those on the right more abundant at term. Few clusters are overrepresented in the 120 day lung but include rapidly dividing cells (cluster 15), AF1 cells (cluster 1) and immature AT1/AT0 cells (cluster 0), which just reach statistical significance. Cluster 0 likely encompasses multiple immature cell types from the proximal airway, since although the AT1 cell marker *AGER* is high on the differentially expressed gene (DEG) list, several surfactant protein genes including *SFTPB* and *SFTPC* are also evident. This immature cluster may also reflect the complexity of epithelial cellularity in the terminal and respiratory bronchioles in adult lung (Basil et al. 2022; Kadur Lakshminarasimha Murthy et al. 2022). In contrast, the adjacent cluster 4 (which does not separate from cluster 0 at a resolution of 0.1) reflects more fully differentiated AT2 cells. Again, the overabundance of erythroid cells (cluster 14) likely reflects an artifact of depletion efficacy.

Clusters that are significantly more abundant in the term lung include: LEC (cluster 20) and mast cells (cluster 19), which just reach statistical significance; VEC (cluster 2); AF2 cells (cluster 6); SVEC/AEC (cluster 12); T cells (cluster 16); macrophages (cluster 10); basal cells (cluster 11) and secretory epithelial cells (cluster 7). The remaining 8 clusters including cluster 13 (chondrocytes), cluster 4 (AT2 cells), cluster 9 (putative goblet cells, based on marker genes *LTF, AGR2, AQP5, CREB3L4* and *SPDEF,* though *MUC5AC* is not annotated in the Oar_v4.0 genome), cluster 3 (a mesenchymal cluster, likely VSMC), cluster 8 (likely SMCF), cluster 18 (ciliated cells), cluster 17 (poorly defined endothelial cells) and cluster 5 (ASMC) were equally abundant at 120 days and term. The tissue from one lamb (AXH141) was observed to have an unusual cell distribution compared to other term samples, with a bias towards epithelial cells, but removal of this sample from the merged analysis did not alter the clusters that were overrepresented at term compared to 120 days, it merely slightly reduced the obs_log2FD values for basal cells.

In summary, the major changes in cell abundance between 120 days and term in proximal lung are i) a further reduction in rapidly dividing cells and fibroblasts and in poorly differentiated AT1/AT0 cells, likely due to an increase in alveolar type cell differentiation, and ii) a marked increase in secretory epithelial cells and basal cells, together with increases in several immune cell types including macrophages, T cells and mast cells, and in several endothelial cell populations. These changes broadly coincide with those described during the canalicular and saccular phases of human lung development. Also notable is the activation of the adaptive immune response during this time interval.

### Evolution of cell type abundance through gestation in WT distal lung

#### A. Distal lung development between 80 and 120 days of gestation coincides with an increase in immature ciliated cells, alveolar type cells and vascular endothelial cells

**Fig. 3:**
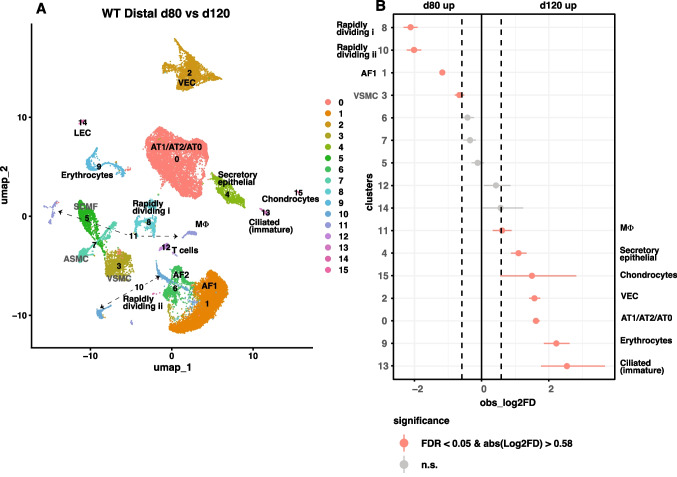
Single cell RNA-seq shows developmental changes in cell abundance between 80 and 120 days in WT sheep distal lung. **A**. UMAP plot of merged data from 8 donors (4 each at 80 days and 120 days) and identifies 16 clusters by differential gene expression profiles, each named by cell type. Abbreviations: in Fig. 1 legend. **B**. Single cell proportions test comparing day 80 to day 120 shows significant overrepresentation of cells in 80 day clusters on the left and in 120 day clusters on the right

WT distal lung at 80 days and 120 days were compared at a resolution of 0.2 yielding sixteen clusters and cluster assignments made based on the marker gene lists (Fig. 3A, Tables S3, Si, Sii). As expected, the cluster abundance was quite different from that of proximal lung (Fig. 1A), however, again it was challenging to unequivocally identify some of the mesenchymal cell clusters (cluster 1, 3, 5, 6, 7) probably due to their immaturity. Endothelial cells are found in clusters 2 and 14, while immune cells are found in clusters 11 and 12, erythrocytes in cluster 9 and endoderm-derived epithelial cells in clusters 0, 4, 13, 14 and 15. An additional feature of these earlier timepoints are several clusters (8 and 10) of rapidly dividing cells. Cluster 10 also has some markers of mesenchymal cells, suggesting these may be rapidly dividing AF1 cells. Clusters 8 and 10 are the most overrepresented clusters in 80 days distal lung when comparing the distribution of cells between 80 and 120 days in a single cell proportions test (Fig. 3B). Also more abundant at 80 days are AF1 cells and a mesenchymal cell cluster (cluster 3) that is likely VSMC but does not have a unequivocal identity based on marker genes in comparison with two other mesenchymal clusters (cluster 5 and cluster 7). More clusters are overrepresented at 120 days, on the right side of Fig. 3B, including immature ciliated cells (cluster 13); immature AT1/AT2/AT0 (cluster 0); VECs (cluster 2); chondrocytes (cluster 15), albeit with a very large error bar showing donor tissue variation; immature secretory epithelial cells (cluster 4); and alveolar macrophages (cluster 11) which just reaches statistical significance. However, cluster 11 includes two separate clusters in the UMAP space, one of which may be macrophages and the other possibly additional myeloid cells. The overrepresentation of erythroid cells (cluster 9) is again likely an artifact of the depletion protocol. There was no difference in the distribution of cells in cluster 6 (AF2 cells), cluster 7 (likely ASMC), cluster 5 (likely SCMF), cluster 12 (T cells) or cluster 14 (LEC).

In summary, the major changes in cell abundance between 80 and 120 days in distal lung are i) a reduction in rapidly dividing cell populations and fibroblasts and ii) an increase in several epithelial cell types including immature ciliated cells, alveolar-type cells, chondrocytes and secretory epithelial cells, and VECs. These changes are broadly consistent with human lung development through the late pseudoglandular and canalicular phases, as described above for the proximal lung tissues.

#### B. Distal lung development between 120 days of gestation and term coincide with an increase in abundance of pericytes, macrophages, T cells, and smooth muscle cells

**Fig. 4:**
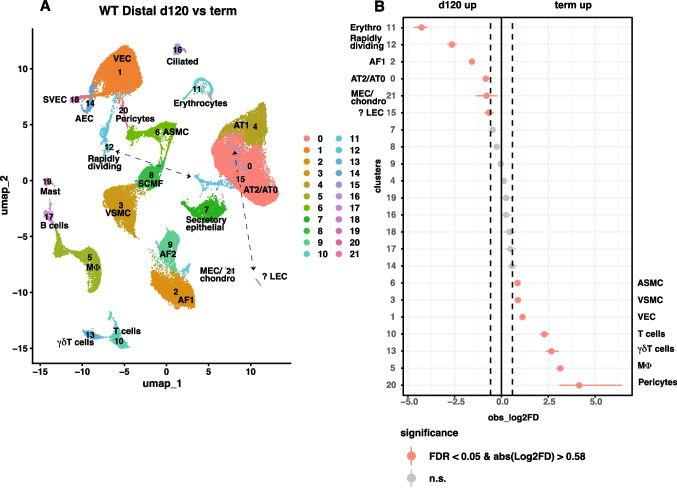
Single cell RNA-seq shows developmental changes in cell abundance between 120 days and term in WT sheep distal lung. **A**. UMAP plot of merged data from 10 donors (4 at 120 days and 6 at term) and identifies 22 clusters by differential gene expression profiles, each named by cell type. Abbreviations as in Fig. 1 legend. **B**. Single cell proportions test comparing day 120 to term shows significant overrepresentation of cells in 120 day clusters on the left and in term clusters on the right

WT distal lung at 120 days and at term were compared using a resolution of 0.2, which yielded 22 clusters (Fig. 4A, Tables S4, Si, Sii). Mesenchymal cells were found in clusters 2, 3, 6, 8 and 9 with a clearer cell-type identity than in the earlier time point comparison. Endothelial cells were in clusters 1, 14, 15 and 18, immune cells in clusters 5, 10, 13, 17 and 19, erythrocytes in cluster 11 and epithelial cells in clusters 0, 4, 7, 16 and 21. One cluster of rapidly dividing cells was also identified (cluster 12) and was more abundant at 120 days than at term (Fig. 4B). Other clusters with a greater abundance of cells at 120 days than at term included: AF1 cells in cluster 2; AT2/0 (cluster 0), chondrocytes (cluster 21) and likely LEC (cluster 15) though the latter two clusters only just reached statistical significance. Erythrocytes were also more abundant at 120 days, reflecting incomplete efficacy of the depletion protocol. Among clusters that were more abundant at term were pericytes (cluster 20, albeit with a large error bar); alveolar macrophages (cluster 5); gamma-delta T cells (cluster 13); other T cells (cluster 10); VEC (cluster 1), likely VSMC (cluster 3); and ASMC (cluster 6). There was no difference in the abundance of 120 day and term cells in cluster 7 (secretory epithelial cells), cluster 8 (SMCF), cluster 9 (AF2 cells), cluster 4 (AT1 cells), cluster 19 (mast cells), cluster 16 (ciliated cells), cluster 18 (SVEC), cluster 17 (B cells) or cluster 14 (AEC).

In summary, the major changes in cell abundance between 120 days and term in distal lung are i) a reduction in rapidly dividing cells and fibroblasts and in immature alveolar type (AT2/AT0) cells, and ii) and increase in pericytes, several immune cell types including macrophages, several types of T cells, VECs, and vascular and airway smooth muscle cells. These alterations share many features of the canalicular and saccular phases of human lung development, as described above for the proximal lung. Again, the activation of the adaptive immune response during this time interval is notable.

### Comparative evolution of cell type abundance through gestation in WT and CF proximal lung

Next, we compared the changes in cell type abundance in WT lung with those of the lungs from age-matched CF (*CFTR*^*−/−*^) lambs, as described previously (Leir et al. 2025). All CF datasets are shown at a resolution of 0.2. Our goal was to identify potential impacts of loss of functional CFTR on the cellularity of the developing lung, since there is little evidence for pathology in the CF lung before birth. However, we observed a cellular imbalance at each timepoint when directly comparing CF and WT scRNA-seq data (Leir et al. 2025).

First, comparing changes in cell abundance between 80 and 120 days in CF proximal lung (Fig. S2, Table S5) we see rapidly dividing cell populations, SMCF, AF1 and secretory epithelial cells to be overrepresented at 80 days. At 120 days, both T and B cells are more abundant, as are AEC, VEC and a rapidly dividing endothelial cell population. Also overrepresented at 120 days are several epithelial cell populations including TP63-positive basal cells and AT2/AT1/AT0 cells, while ASMC and ciliated cells both just reach statistical significance. Next, comparing changes in cell abundance between 120 days and term in CF proximal lung (Fig. S3, Table S6), among overrepresented clusters at 120 days are rapidly dividing cells, AF1 cells, several epithelial cell types including AT2/AT0 cells and goblet cells, also mast cells. Only 4 clusters show increased abundance at term, specifically ciliated cells, macrophages (alveolar), and both vascular and systemic venous endothelial cells.

Contrasting these data from the CF proximal lung with that of WT sheep (Table 3A), we see that many of the developmental changes in CF animals mirror those in the WT. Minor differences are seen between day 80 and day 120, specifically T cells are more abundant at day 80 only in WT as are SMCF in CF animals. At 120 days overabundant T cells, immature B-cells and ASMC are only evident in CF animals, which also lack the increased abundance of secretory epithelial cells, chondrocytes and PNEC evident in WT animals. More extensive differences are seen between CF and WT animals comparing day 120 and term. Although at 120 days CF animals uniquely have more mast cells and goblet cells than at term, they do not show the comparative overabundance of AF2 cells, LEC, T cells, basal cells and secretory epithelial cells seen in term WT animals. Instead, they show overrepresentation of ciliated cells, consistent with our previous observations (Leir et al. 2025). Together these results suggest that although proximal lung development and differentiation is similar in the CF and WT fetal sheep before 120 days, later in gestation the CF lung may show significant alterations particularly in epithelial cell populations.

**Table 3:** Comparison of changes in cluster representation in CF and WT lungs through gestation

**A**	**Proximal CF**	**Proximal WT**
d80 vs d120: d80 up	Rapidly dividing general Rapidly dividing AT cells SMCF AF1 cells	Rapidly dividing general AF1 cells T cells
d80 vs d120: d120 up	T cells AEC Basal cells Rapidly dividing endothelial VEC B cells (immature) AT1/AT2/AT0 cells ASMC Ciliated cells	Secretory epithelial AT1/AT2 cells VEC Chondrocytes Ciliated cells SVEC/AEC PNEC Basal cells
d120 vs term: d120 up	Rapidly dividing AF1 cells AT1/AT2/AT0 cells Mast cells Club cells	Rapidly dividing AF1 cells AT1/AT0 cells
d120 vs term: term up	SVEC VEC Macrophages (alveolar) Ciliated cells	LEC Mast cells VEC AF2 cells SVEC/AEC T cells Macrophages (alveolar) Basal cells Secretory epithelial cells
**B**	**Distal CF**	**Distal WT**
d80 vs d120: d80 up	Rapidly dividing cells SMCF AF1	Rapidly dividing cells Rapidly dividing AF cells AF1 cells VSMC
d80 vs d120: d120 up	Macrophages (alveolar) ASMC VEC Rapidly dividing VEC AT1 cells Immature B cells T cells	Macrophages (alveolar) Secretory epithelial cells Chondrocytes VEC AT1/AT2/AT0 cells Immature ciliated cells
d120 vs term: d120 up	Rapidly dividing cells AF1 cells Mast cells AT2 cells	Rapidly dividing cells AF1 cells AT2/AT0 cells Chondrocytes LEC
d120 vs term: term up	VEC Pericytes LEC Ciliated cells Macrophages (alveolar)	ASMC VSMC T cells γδ T cells Macrophages (alveolar) Pericytes

Equivalent comparisons of cell populations were then performed on distal lung from CF sheep. First, comparing changes in cell abundance between 80 and 120 days (Fig. S4, Table S7), again rapidly dividing cells, and two mesenchymal cell populations, SCMF and AF1 cells were more abundant at 80 days. In contrast, multiple immune cell types including T cells, B cells and alveolar macrophages were more abundant at 120 days, as were AT1 cells, VECs together with a rapidly dividing cell cluster with VEC marker genes, and ASMCs. Next, comparing 120 day and term distal CF lung (Fig. S5, Table S8), clusters that are overrepresented at 120 days include rapidly dividing cells, AF1 cells, mast cells and AT2 cells which just reach statistical significance. At term, macrophages, ciliated cells, LEC, pericytes and VEC are overrepresented.

Comparing these data from the CF distal lung with that of WT sheep (Table 3B) we again see similar profiles. In the day 80 to day 120 comparison, both genotypes show largely equivalent cell types that are more abundant at day 80, though at 120 days, there is less overlap, with immature B cells and T cells being overabundant only in the CF animals, while chondrocytes, secretory epithelial cells and immature ciliated cells are overrepresented in WT. Next, comparing 120 days and term, at 120 days only CF animals show mast cell overrepresentation, while in WT both LEC and chondrocytes are more abundant. At term, as in the proximal lung, the two genotypes show divergence, with VEC, pericytes, LEC and ciliated cells being overabundant compared to 120 days in CF and ASMC, VSMC and T cells (also $\gamma \delta$ T cells) being overabundant in WT animals. Hence, though the CF and WT animals show broadly similar patterns of differentiation in the distal lung, there may be alterations particularly in immune cell compartments, but also among endothelial cell and epithelial cell distributions in the CF animals.

## Discussion

Among our main goals in this study was to throw light on late stage cellular aspects of lung differentiation at time points that are rarely accessible in human. By using the sheep lung as a model, we built upon substantial comparative data from many earlier investigations. We performed separate analysis from two lung regions (Fig. S1), the first at the base of the middle right lung lobe centered on the primary bronchus (proximal) and the second in the most caudal region of the right lobe (distal). Of note the terms proximal and distal here are not based upon airway diameter but rather anatomical location in the lung. The same scRNA-seq dataset used in the current analysis provides the opportunity to compare the cellularity of these two lung regions at the single cell level (Fig. S6). At 120 days of gestation both chondrocytes and basal cells are significantly more abundant in the proximal lung, as are goblet cells, basal cells, SVEC and AF2 cells at term. In contrast several immune cell types including T cells, $\gamma \delta$ T cells and macrophages are more abundant in the distal lung at term. Most cluster identities are based on our previous detailed sheep lung cell characterization (Leir et al. 2025), which were facilitated by LungMap data (Sun et al. 2022) and other publications (He et al. 2022; Sountoulidis et al. 2023).

Although there were some challenges in definitive identification of stromal cell sub-types particularly in the 80 day lung, the general patterns of changes in cell abundance between 80 and 120 days overlapped in proximal and distal lung tissue. In both lung regions, a reduction in several populations of poorly differentiated rapidly dividing cells and fibroblasts was evident, likely reflecting the major expansion of the lung in the earlier stages of development. In both lung regions at 120 days there was an increase in both endothelial cell and epithelial cell compartments, though the specific cell types were different in the two parts. In the proximal lung, VECs and SVECs/AECs increased at 120 days while in the distal lung VECs alone were significantly more abundant. In both cases the changes likely reflect the expansion of blood vessels within the developing lung. Considering epithelial cell populations in the proximal lung, basal, ciliated and secretory epithelial cells together with immature AT1/AT2/AT0 cells, chondrocytes and PNECs were more abundant at 120 days. These observations are consistent with the branching expansion of large and small airways during the canalicular phase. The same epithelial cell types were overrepresented in the distal lung at 120 days, with the exception of basal cells and PNECs (which were not evident at this time point). Further, the gene expression profile of the ciliated cells was less well differentiated. Macrophages also reached statistical significance for overrepresentation in the distal lung at 120 days.

Perhaps most interesting is the ability to track events between 120 days and term, when the lung is prepared for the dramatic change from being fluid-filled in utero to perform gas exchange at birth. In the proximal lung between 120 days and term there is a further significant reduction in rapidly dividing cells and airway fibroblasts, though the apparent decrease in AT1/AT0 cell abundance is likely an artifact of the differentiation of AT2 cells which now populate a separate cluster. More abundant clusters at term include multiple immune cell types, specifically mast cells, T cells and macrophages, also several endothelial cell types including LECs, VECs and SVECs/AECs, and most significantly, secretory epithelial cells and basal cells. Across the same time course in the distal lung, again rapidly dividing cells and airway fibroblast decrease in abundance, while the changes in AT2/AT0 abundance are, as in proximal lung, due to the separation of AT1 and AT2 cells into separate clusters as their differentiation progresses. The profile of more abundant clusters in the distal lung at term diverges from that of proximal lung in that none are epithelial cell types and the most significantly overrepresented clusters are macrophages, T cells and $\gamma \delta$ T cells, while pericytes, VECs, VSMCs and ASMCs are also more abundant. These differences reflect the functional progression of the two parts of the lung at term.

Lastly, we compare the differentiation profiles of proximal and distal lung across the time interval from 80 days, through 120 days to term in WT lambs to those of CF lambs across the same time course (Table 3). In the proximal lung (Table 3A) we see substantial overlap between the two genotypes between 80 and 120 days although T cells and immature B cells are overrepresented only in the CF lung at 120 days, possibly correlating with early immune activation. More divergence is evident when alterations between 120 days and term are compared. Particularly noticeable is the overabundance of ciliated cells at term only in the CF animals, which concurrently lack the overrepresentation of other epithelial cell types that is seen in WT lambs, specifically in basal cells and secretory epithelial cells. In the distal lung (Table 3B), again there is similarity in the differential cluster abundance at 80 days, although again T cells and immature B cells are overabundant at 120 days only in the CF lambs, while immature ciliated cells and secretory epithelial cells are overabundant in the WT lambs. In the 120 day to term comparison, the profiles of overrepresented clusters at term are quite different in the two genotypes with ciliated cells and endothelial cells being more abundant in the CF animals and smooth muscle cells and T cells being overrepresented in WT. Together the results suggest that though the human lung is thought rarely to exhibit a CF disease-associated phenotype at birth, the alterations seen in the CF lambs suggest cellular changes are evident through gestation and at birth. Currently the CF lambs do not survive beyond the perinatal period due to a severe intestinal phenotype. However, once this is alleviated, by genetic or pharmacological correction, it will be possible to perform functional assays on the postnatal CF lamb lung to determine the impact on lung biology and its relevance to human CF lung disease. It remains possible that the CF lung phenotype is more severe in sheep.

## Supplementary Information

Below is the link to the electronic supplementary material.

Supplementary file1 (XLSX 311 KB)

Supplementary file2 (XLSX 308 KB)

Supplementary file3 (XLSX 252 KB)

Supplementary file4 (XLSX 353 KB)

Supplementary file5 (XLSX 331 KB)

Supplementary file6 (XLSX 269 KB)

Supplementary file7 (XLSX 278 KB)

Supplementary file8 (XLSX 281 KB)

Supplementary file9 (PDF 85 KB)

Supplementary file10 (PDF 67 KB)

Supplementary file11 (PDF 38243 KB)

## Data Availability

All sequencing data used in this project are deposited at GEO: GSE281174; (Leir et al. 2025).
