# Supplementary material for: Developmental dynamics of ovine lung in health and cystic fibrosis at single-cell resolution
Source: Funct Integr Genomics. 2026 Jul 31;26(1):213. doi: 10.1007/s10142-026-01981-2 (PMC13424028; doi:10.1007/s10142-026-01981-2)
Supplement: Supplementary file 9 — Supplementary file9 (PDF 85 KB) [file 10142_2026_1981_MOESM9_ESM.pdf]

## Table Si

A. Marker genes for clusters in 80 day to 120 day proximal lung development

| CLUSTER # | CELL IDENTITY | MARKER PROCESSES | MARKER GENES | |
|---|---|---|---|---|
| **8** | Rapidly dividing cells | Cell cycle/division<br><br>DNA replication | *STMN1, TPX2*<br><br>*TOP2A, PCNA* | More abundant 80 days |
| **14** | Rapidly dividing alveolar fibroblast-like (AF1-like) | Fibroblast structure/function<br><br>Cell proliferation | *CA3, PPP1R14A*<br><br>*TMSB4X, TPT1, ZC3H10* | |
| **0** | AF1 cells | | *CA3, TCF21* and *PPP1R14A1* | |
| **15** | Immune: T cell | | *CD3E, CD3G* | |
| | | | | |
| **11** | Basal cells | | *KRT15, KRT5, TP63* | More abundant 120 days |
| **19** | PNEC | | *NRXN1* | |
| **10** | Erythroid | | *HBA1 HBG, HBM* | |
| **13** | SVEC/AEC | | *PECAM1, PLVAP, VWF, ACKR1* | |
| **16** | Ciliated | | *TSPAN1, CAPSL, FOXJ1* | |
| **9** | Chondrocytes | | *COL9A1, A2, A3, COL2A1, HAPLN1, ACAN, CYTL1, CHAD* | |
| **2** | VEC | | *PECAM, CLEC14A* | |
| **7** | Secretory epithelial cells | | *SCGB3A2, TFF3, MUC1, EHF* | |

B. Marker genes for clusters in 120 day to term proximal lung development

| CLUSTER # | CELL IDENTITY | MARKER PROCESSES | MARKER GENES | |
|---|---|---|---|---|
| **15** | Rapidly dividing cells | Cell cycle/division<br><br>DNA replication | *STMN1, TPX2*<br><br>*TOP2A, PCNA* | More abundant 120 days |
| **1** | AF1 cells | | *CA3, TCF21* and *PPP1R14A1* | |
| **0** | Immature AT1/AT2/AT0 | | *AGER, SFTPB, SFTPC* | |
| **4** | AT2 | | *SFTPC, SFTPB, SLC34A2, SFTPA, SFTPD* | |
| | | | | |
| **20** | LEC | | *MMRN1, PROX1* | |

| | | | |
|---|---|---|---|
| **19** | Mast cells | | *KIT, LTC4S, TPSB2* |
| **2** | VEC | | *PECAM, CLEC14A* |
| **6** | AF2 | | *MFAP5, PI16, OGN, CCDC80, COL3A1* |
| **12** | SVEC/AEC | | *PECAM1, PLVAP, VWF, ACKR1* |
| **16** | T cells | | *CD3E, CD3G* |
| **10** | Macrophages | | *ALOX5AP, CD68, SPI1, CTSS* |
| **11** | Basal cells | | *KRT15, KRT5, TP63* |
| **7** | Secretory epithelial cells | | *SCGB3A2, TFF3, SPDEF, EHF, SOX2* and *SOX9* |

More abundant term

C. Marker genes for clusters in 80 day to 120 day distal lung development

| **CLUSTER #** | **CELL IDENTITY** | **MARKER PROCESSES** | **MARKER GENES** |
|---|---|---|---|
| **8** | Rapidly dividing cells | Cell cycle/division<br><br>DNA replication | *STMN1, TPX2,CENPW*<br><br>*TOP2A, PCNA* |
| **10** | Rapidly dividing AF1-like | Fibroblast structure/function<br><br>Cell proliferation | *CA3, PPP1R14A*<br><br>*TUBB, CENPF* |
| **1** | AF1 cells | | |
| **3** | VSMC | | |
| | | | |
| **13** | Ciliated cells | | *TSPAN1, CAPSL, FOXJ1* |
| **0** | Immature AT1/AT2/AT0 cells | | *AGER, SLC34A2,, SFTPC* |
| **2** | VEC | | *PECAM, CLEC14A* |
| **15** | Chondrocytes | | *COL9A3, COL2A1, HAPLN1, ACAN, CYTL1, CHAD* |
| **4** | Immature secretory epithelial cells | | *TFF3, KRT8, KRT19, CLDN10, KLF5,* EHF |
| **11** | Macrophages | | |
| **9** | Erythroid | | |

More abundant 80 days

More abundant 120 days

D. Marker genes for clusters in 120 day to term distal lung development

| **CLUSTER #** | **CELL IDENTITY** | **MARKER PROCESSES** | **MARKER GENES** |
|---|---|---|---|
| **12** | Rapidly dividing cells | Cell cycle/division<br><br>DNA replication | *STMN1, TPX2*<br><br>*TOP2A, PCNA* |
| **2** | AF1 cells | | *CA3*, *TCF21* and *PPP1R14A1* |
| **0** | Immature AT2/AT0 | | *SFTPC, SFTPB, SLC34A2, SFTPA, SFTPD* |
| **21** | Chondrocytes | | *COL9A3, COL2A1, HAPLN1, ACAN, CYTL1, CHAD* |
| **15** | LEC | | *MMRN1* |
| **11** | Erythroid | | |
| | | | |
| **20** | Pericytes | | *HIGD1B, FAM162B, COX412* |
| **5** | Alveolar macrophages + monocytes | | *CD68, ALOX5AP, SPI1, CTSS* |
| **13** | gamma-delta T cells | | *CD7, TRDC* |
| **10** | T cells | | *CD3E, CD3G* |
| **1** | VEC | | |
| **3** | VSMC | | *SERPINE2* |
| **6** | ASMC | | *GUCY1B3, CALD1, ACTA2, MYH11, MYLK* |
