# Supplementary material for: Developmental dynamics of ovine lung in health and cystic fibrosis at single-cell resolution
Source: Funct Integr Genomics. 2026 Jul 31;26(1):213. doi: 10.1007/s10142-026-01981-2 (PMC13424028; doi:10.1007/s10142-026-01981-2)
Supplement: Supplementary file 10 — Supplementary file10 (PDF 67 KB) [file 10142_2026_1981_MOESM10_ESM.pdf]

**Official Gene symbol**:	**Full gene name**:
STMN1	stathmin 1
TPX2	TPX2 microtubule nucleation factor
TOP2A	DNA topoisomerase II alpha
PCNA	proliferating cell nuclear antigen
CA3	carbonic anhydrase 3
PPP1R14A	protein phosphatase 1 regulatory inhibitor subunit 14A
TMSB4X	thymosin beta 4 X-linked
TPT1	tumor protein, translationally-controlled 1
ZC3H10	zinc finger CCCH-type containing 10
TCF21	transcription factor 21
KRT15	keratin 15
TP63	tumor protein 63
NRXN1	neurexin 1
PECAM	platelet endothelial cell adhesion molecule 1
PLVAP	plasmalemma vesicle associated protein
VWF	Von Willebrand factor
ACKR1	atypical chemokine receptor 1 (duffy blood group)
TSPAN1	tetraspanin
CAPSL	calcyphosin like
FOXJ1	forkhead box J1
COL9A1	collagen 9A1
HAPLN1	hyaluronan and proteoglycan link protein 1
ACAN	aggrecan
CYTL1	cytokine-like 1
CHAD	chondroadherin
CLEC14A	C-type lectin domain containing 14A
SCGB3A2	secretoglobin family 3A member 2
TFF3	trefoil factor 3
MUC1	mucin 1
EHF	ETS homologous factor
AGER	advanced glycosylation end-product specific receptor
SFTPC	surfactant protein C *SFTPC*
SLC34A2	solute carrier family 34 member A2
MMRN1	multimerin 1
PROX1	prospero homeobox 1
KIT	KIT proto-oncogene, receptor tyrosine kinase
LTC4S	leukotriene C4 synthase
TPSB2	tryptase beta-2
ALOX5AP	arachidonate 5-lipoxygenase activating protein
SPI1	Spi-1 proto-oncogene
CTSS	cathepsin S
SPDEF	SAM pointed domain containing ETS transcription factor
SOX2	SRY-box transcription factor 2
LTF	lactotransferrin
AGR2	anterior gradient 2, protein disulphide isomerase family member
AQP5	aquaporin 5
CREB3L4	cAMP responsive element binding protein 3 like 4
TUBB	tubulin beta class 1
CENPW	centromere protein w
CLDN10	claudin 10
KLF5	krüppel-like factor 5
HIGD1B	HIG1 hypoxia inducible domain family member 1B
FAM162B	family with sequence similarity 162 member B
COX412	cytochrome C oxidase subunit 42
TRDC	T cell receptor delta locus
SERPINE2	serpin family E member 2
GUCY1B3	guanylate cyclase 1 soluble subunit beta 1
CALD1	caldesmon 1
ACTA2	actin alpha 2, smooth muscle
MYH11	myosin heavy chain 11
MYLK	myosin light chain kinase

Table Sii. Official gene symbols and full gene names
