# Supplementary material for: Developmental dynamics of ovine lung in health and cystic fibrosis at single-cell resolution
Source: Funct Integr Genomics. 2026 Jul 31;26(1):213. doi: 10.1007/s10142-026-01981-2 (PMC13424028; doi:10.1007/s10142-026-01981-2)
Supplement: Supplementary file 11 — Supplementary file11 (PDF 38243 KB) [file 10142_2026_1981_MOESM11_ESM.pdf]

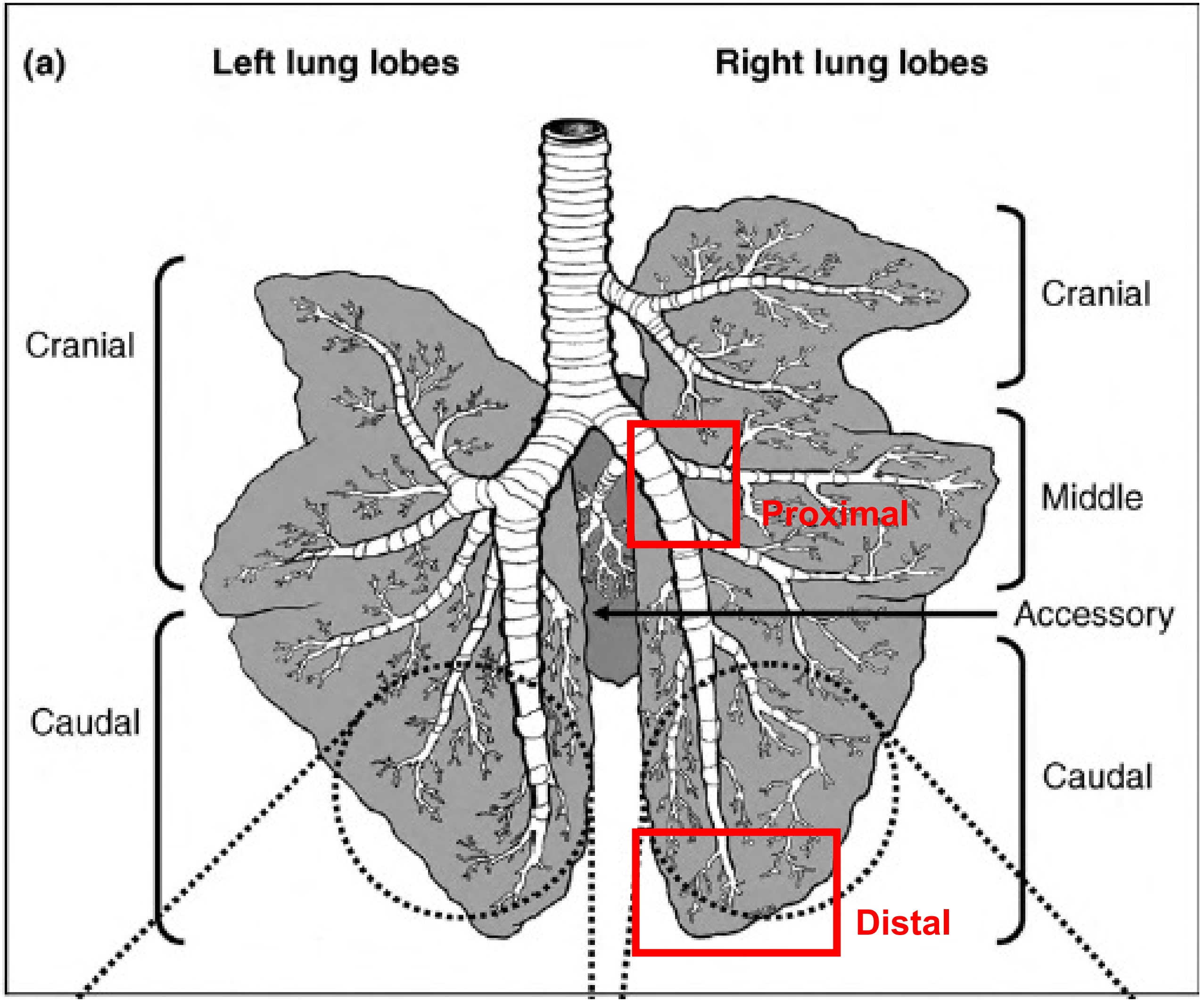

- Image from 10.1016/j.ddmod.2009.12.002

Figure S1

Proximal and distal lung tissue collection sites shown as red boxes.

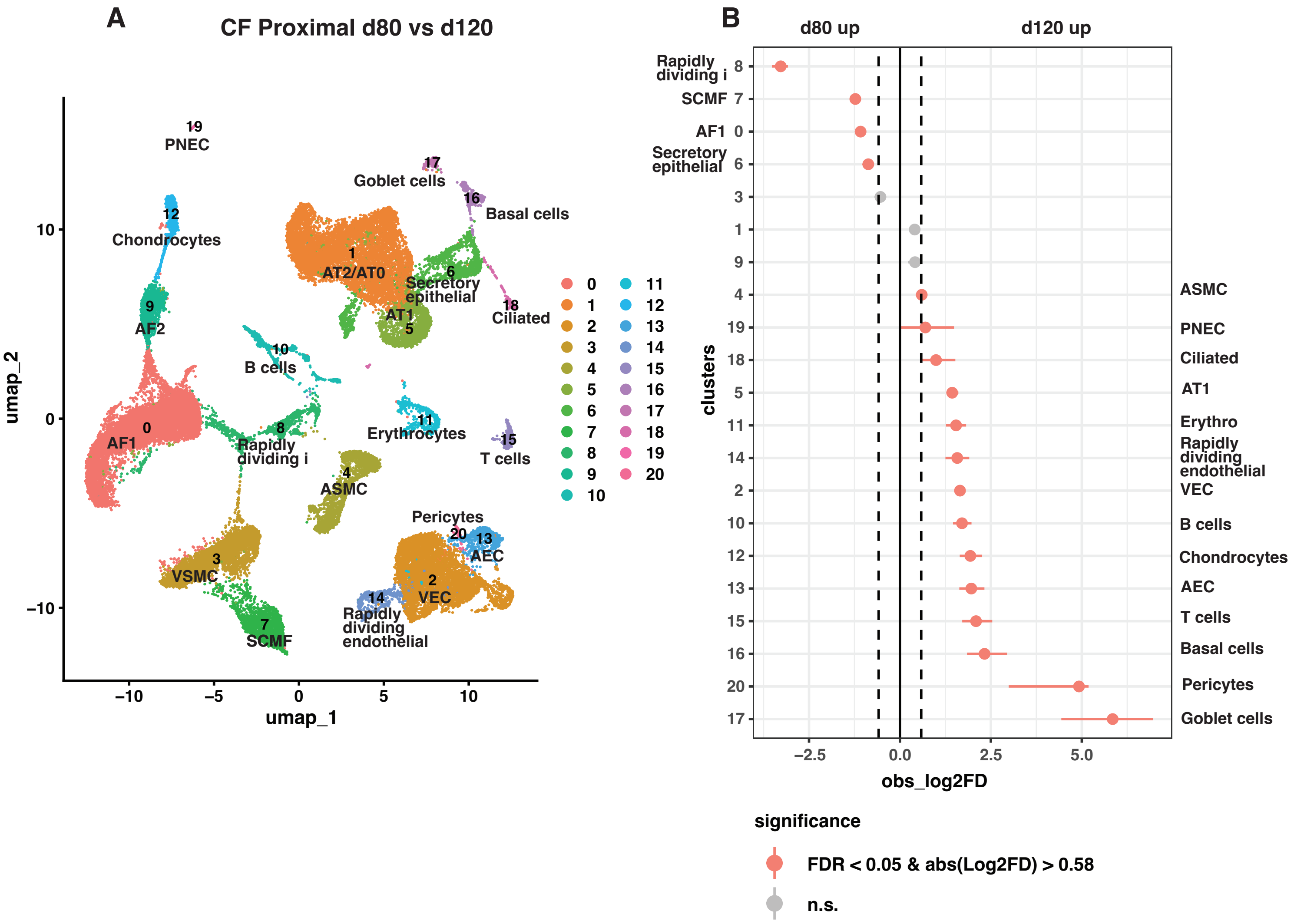

Figure S2

Single cell RNA-seq shows developmental changes in cell abundance between 80 days and 120 days in CF sheep proximal lung. A. UMAP plot of merged data from 10 donors (3 at 80 days and 7 at 120 days) and identifies 21 clusters by differential gene expression profiles, each named by cell type. Abbreviations: AF1/2, alveolar fibroblasts (1/2); AT1/2/0, alveolar type 1/2/0; VEC, vascular endothelial cells; VSMC, vascular smooth muscle cells; ASMC, airway smooth muscle cells; SCMF, secondary crest myofibroblast; MEC, myoepithelial cells; AEC arterial endothelial cells; PNEC, neuroendocrine cells. B. Single cell proportions test comparing day 80 to day 120 shows significant overrepresentation of cells in 80 day clusters on the left and in 120 day clusters on the right.

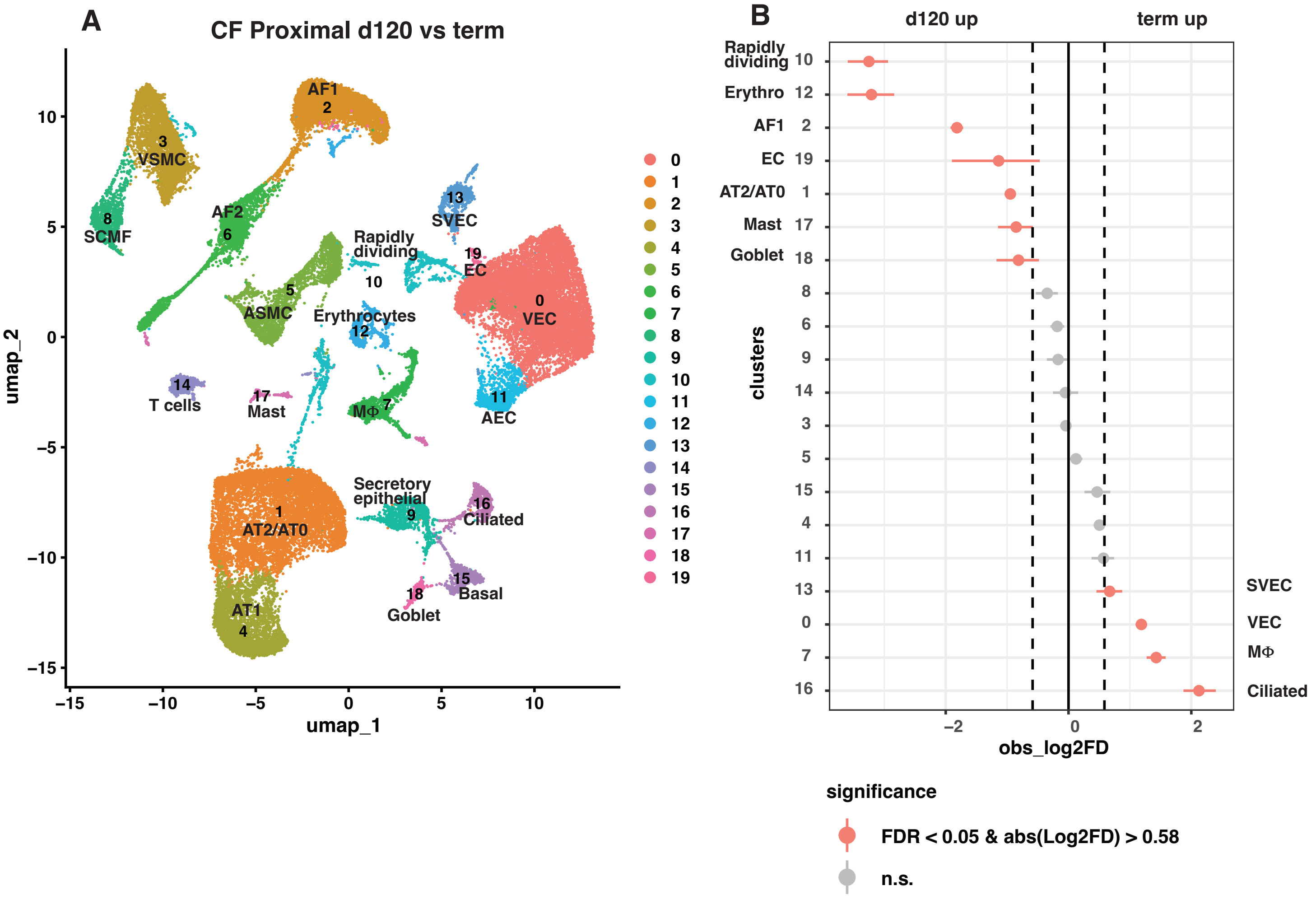

Figure S3

Single cell RNA-seq shows developmental changes in cell abundance between 120 days and term in CF sheep proximal lung. A. UMAP plot of merged data from 9 donors (4 at 120 days and 5 at term) and identifies 20 clusters by differential gene expression profiles, each named by cell type. Abbreviations as defined in Fig. S2 legend. B. Single cell proportions test comparing day 120 and term shows significant overrepresentation of cells in 120 day clusters on the left and in term clusters on the right.

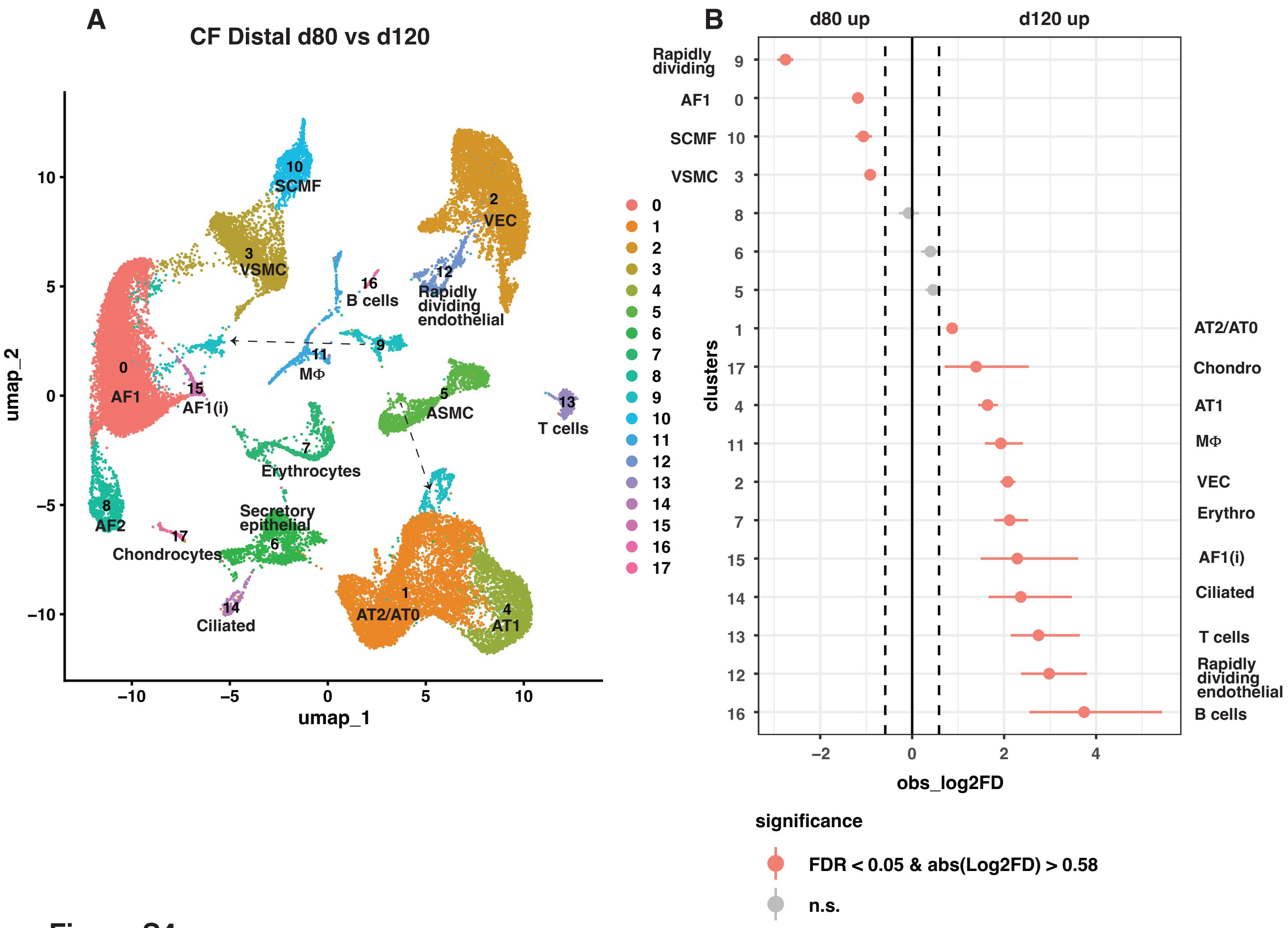

Figure S4

Single cell RNA-seq shows developmental changes in cell abundance between 80 days and 120 days in CF sheep distal lung. A. UMAP plot of merged data from 10 donors (3 at 80 days and 7 at 120 days) and identifies 18 clusters by differential gene expression profiles, each named by cell type. Abbreviations as in Fig. S2 legend. B. Single cell proportions test comparing day 80 to day 120 shows significant overrepresentation of cells in 80 day clusters on the left and in 120 day clusters on the right.

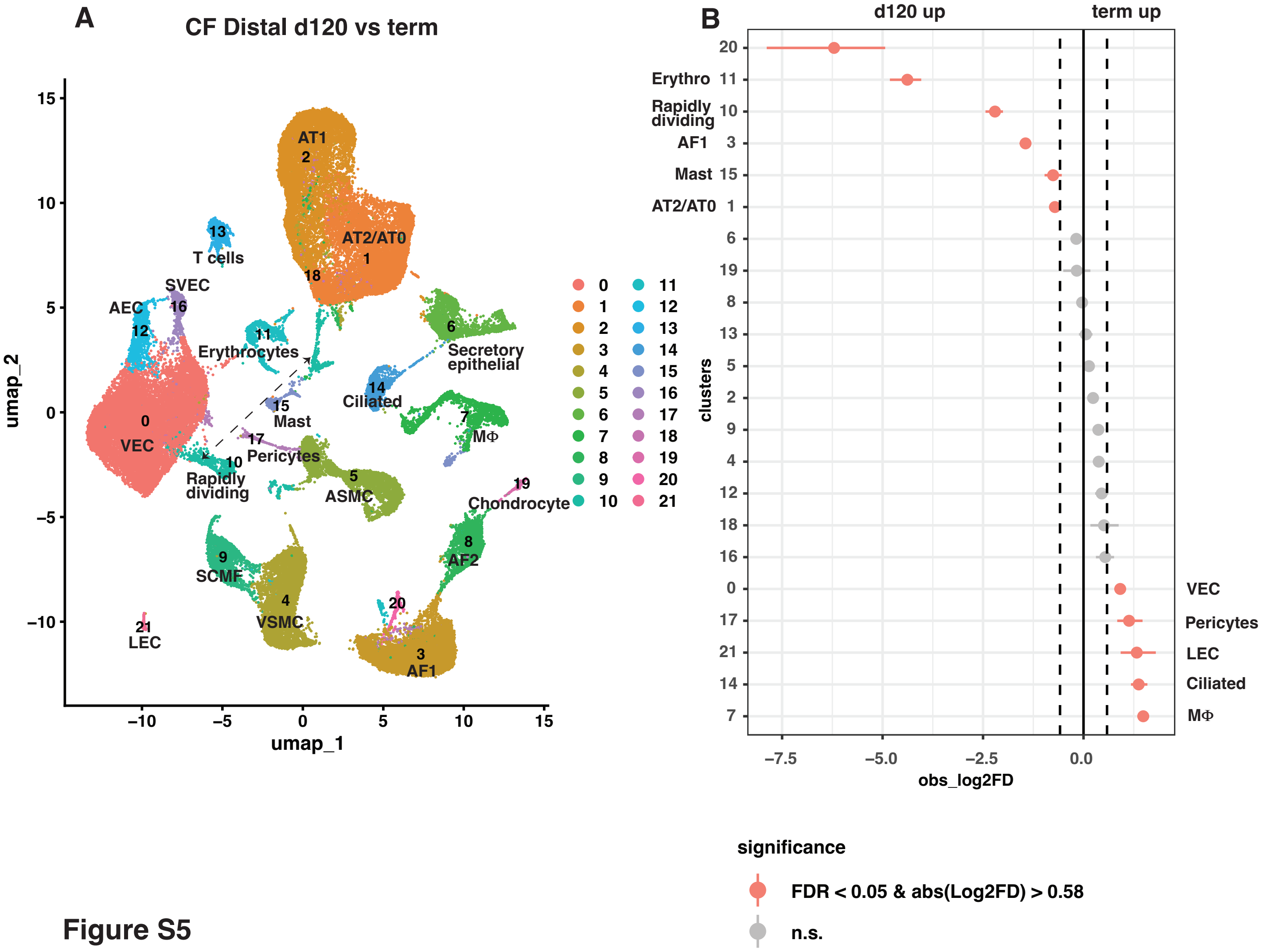

Figure S5

Single cell RNA-seq shows developmental changes in cell abundance between 120 days and term in CF sheep distal lung. A. UMAP plot of merged data from 10 donors (4 at 120 days and 6 at term) and identifies 22 clusters by differential gene expression profiles, each named by cell type. Abbreviations as defined in Fig. S2 legend. B. Single cell proportions test comparing day 120 and term shows significant overrepresentation of cells in 120 day clusters on the left and in term clusters on the right.

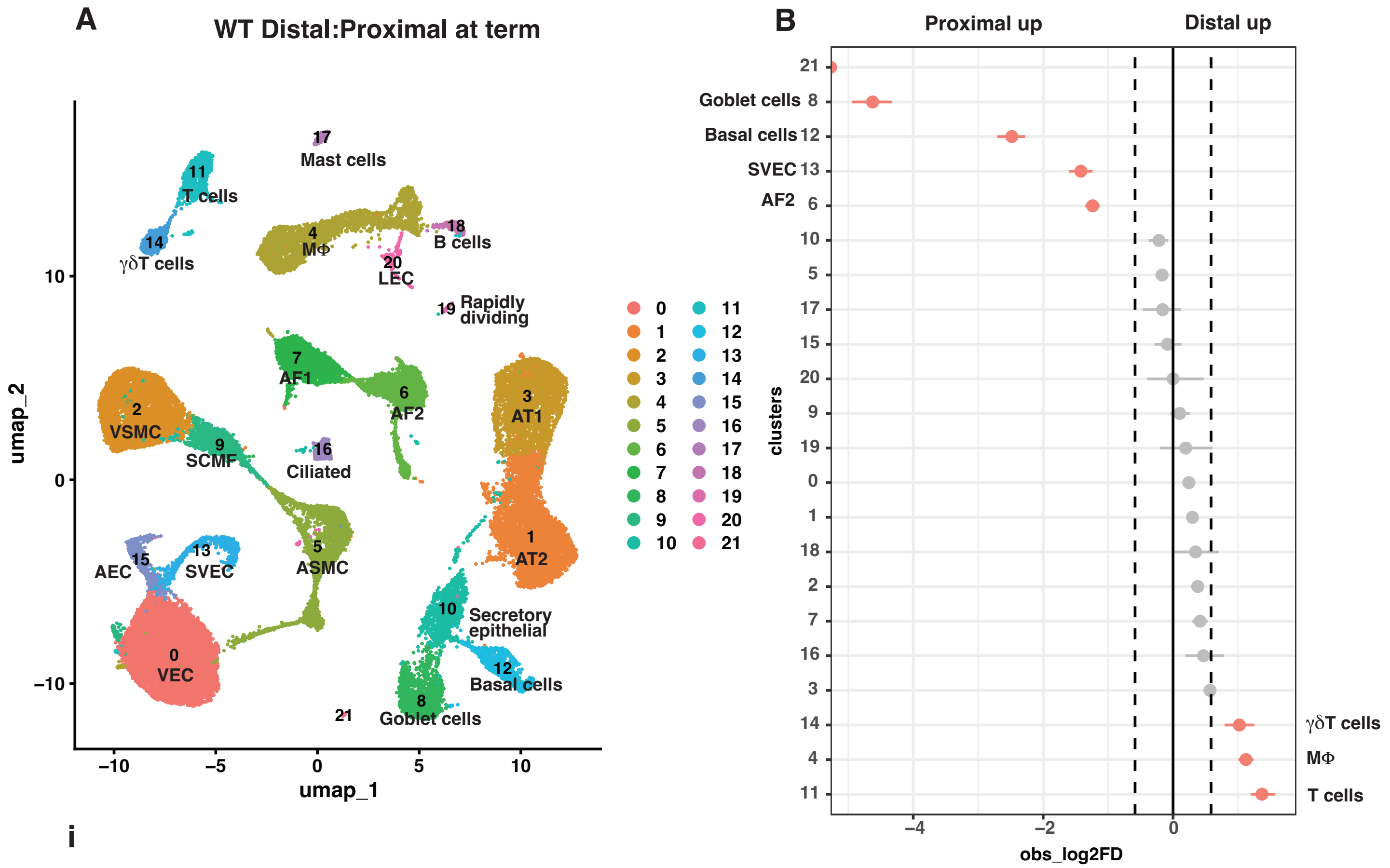

i

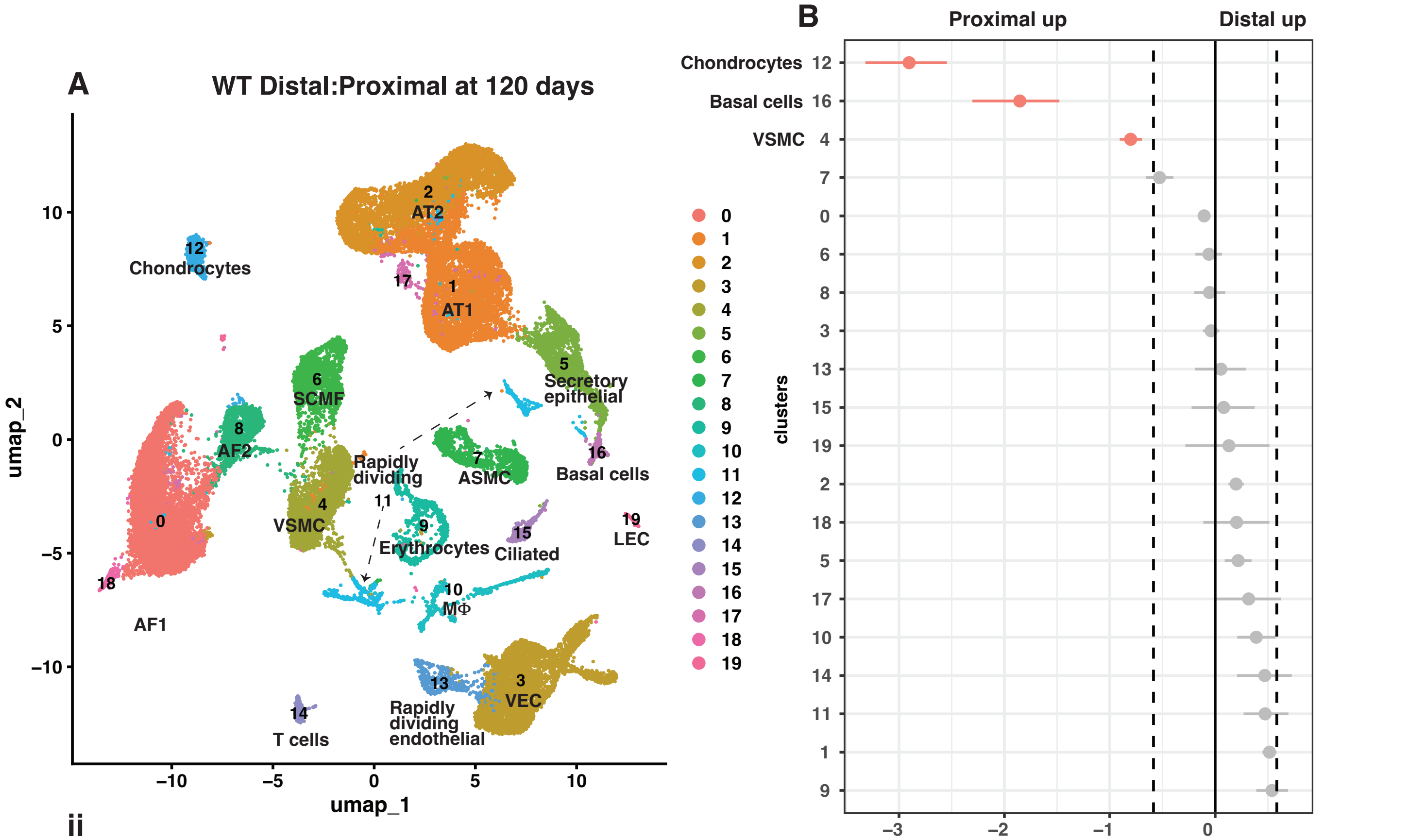

ii

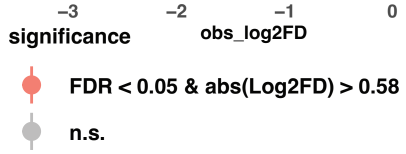

Figure S6

ScRNA-seq shows differences in cell abundance between proximal and distal WT lung at i) term and ii) 120 days.
